# Supplementary material for: Inferring country-specific import risk of diseases from the world air transportation network
Source: PLoS Comput Biol. 2024 Jan 24;20(1):e1011775. doi: 10.1371/journal.pcbi.1011775 (PMC10843136; doi:10.1371/journal.pcbi.1011775)
Supplement: S1 Text — Supplementary Note A: Origin-destination data (“Global Transnational Mobility Dataset”). Supplementary Note B: Symmetrized flows. Supplementary Note C: On the overestimation of low import probabilities. Supplementary Note D: Disease arrival time analysis. Table A. Filtering criteria for the log-cases fit to extrapolate the arrival times tA. A country is excluded if (C0:) the detection is too sparse before peak-0 (less than 6 weeks of data), (C1:) the number of cases at peak-0 is below 30 (otherwise the signal is too noisy), (C2:) the extrapolated arrival time is before the WHO-outbreak date. N is the number of countries for which case data could be generated. NC0 and NC1 are the countries that pass criteria C0 and C1. NC0 & C1 and NC0 & C1 & C2 are the numbers of countries that pass multiple criteria. Table B. Disease and SARS-CoV-2 Variant outbreak information and WAN date. For each disease/variant the outbreak country and the date of the WAN used to compute the import probability estimates with the different models is displayed. Note that we only have the WAN from the years 2014 and 2019 in a yearly resolution and from 2020–2022 in monthly resolution. We repeated the analysis for COVID with the WAN from the month 2020–01-01, instead of using the yearly WAN from 2019, which gave comparable results. Fig A. Import probability dependence on the geographic distance (A), the effective distance (B) and the geographic path distance (C). The orange line represents the median and C(x, y) is the correlation between the two measures either log-transformed or not. The geographic distance between countries is averaged over all airport pairs. The geographic path distance is the geographic distance along the shortest path derived from the WAN using deff, i.e. it is a combination of geographic and network information. The axis scale corresponds to the one with the highest correlation, i.e. log-log for distance and path distance (A, C) and y-log for the effective distance (B). Fig [file pcbi.1011775.s001.pdf]

Supplementary Information
“Inferring country-specific import risk of diseases from the world air transportation network”

Pascal P. Klamser[1,2], Adrian Zachariae[1,2], Benjamin F. Maier[1,2,3,4], Olga Baranov[5,6], Clara Jongen[1,2], Frank Schlosser[1,2], Dirk Brockmann[1,2,7,*],

[1]Department of Biology, Institute for Theoretical Biology, Humboldt-Universität zu Berlin, Berlin, Germany
[2]Robert Koch Institute, Berlin, Germany
[3]DTU Compute, Technical University of Denmark, Kongens Lyngby, Denmark
[4]Copenhagen Center for Social Data Science, University of Copenhagen, Copenhagen, Denmark
[5]Division of Infectious Diseases and Tropical Medicine, University Hospital, LMU Munich, Munich, Germany
[6]German Center for Infection Research (DZIF), Partner Site Munich, Munich, Germany
[7]Center Synergy of Systems (SynoSys), Center for Interdisciplinary Digital Sciences, Technische Universität Dresden, Dresden, Germany
[*]Corresponding author E-mail: dirk.brockmann@tu-dresden.de

Supplementary Note A: Origin-destination data (“Global Transnational Mobility Dataset”)

We use the “Global Transnational Mobility Dataset” (1, 2) as a reference data set of the import probabilities. It is a combination of the World-Air-Transportation-Origin-Destination (WOD) data set from the company SABRE, and cross-boarder visits (CBV) from the UNWTO (World Tourism Organization). The WOD has in contrast to the WAN the real number of passengers from their origin airport to their final destination that booked their tickets via SABREs global distribution system (GDS). The WTO data is based on cross border visits that include an overnight stay of non-residents, thus the backflow of residents in the country is not monitored. The study (1) processed and combined the two data sets by:

1. decompose WOD in trend-, seasonal- and noise-component and only use the **trend** component timeseries :

$$T_{\text{WODall},ij} = T_{\text{WOD},ij} + T_{\text{WODseason},ij} + T_{\text{WODnoise},ij}$$

2. symmetrize the tourism flow matrix (to account for returning residents):

$$\hat{T}_{\text{WTO},ij} = T_{\text{WTO},ij} + T_{\text{WTO},ji}$$

3. correct the WOD data since it underestimates the mobility flow for close countries (the mobility on land or water is missing):

$$\hat{T}_{\text{WOD},ij} = \left(\frac{d(i,j)}{d_{max}}\right)^{1/c} T_{\text{WOD},ij}$$

   with $c \approx 6.8$, $d(i,j)$ as the distance between countries i and j and d_{max} as the maximal distance between all countries. I.e., the closer two countries, the stronger the correction and the connection of the two farthest countries is not corrected.

4. combine the 2 data sets by the following rules: if only one data sets provides info on the connection, take this one, otherwise take the larger flow

$$\hat{T}_{ij} = \begin{cases} \hat{T}_{\text{WTO},ij} & \text{if } \hat{T}_{\text{WOD},ij} = \emptyset \\ \hat{T}_{\text{WOD},ij} & \text{if } \hat{T}_{\text{WTO},ij} = \emptyset \\ \max(\hat{T}_{\text{WOD},ij},\ \hat{T}_{\text{WTO},ij}) & \text{otherwise} \end{cases} \quad \textbf{(A)}$$

Note that the reference data set $\hat{T}_{ij}$ is possibly an overestimation because the WOD data is increased for short connections. The origin to final-destination data from SABRE is derived from bookings via its GDS. However, SABRE only had about 31% market share in 2014 of all GDS‘ (Global Distribution System) (3) and an increasing number of bookings were not done via GDS but directly via the airline company (e.g. about 30% of all Lufthansa flights were booked directly with an increasing trend (4)). The WTO data is limited to overnight stays, i.e. private accommodations are not captured, also tending to underestimate the passenger flow, especially for long-range connections where passenger transport is dominated by airplanes. Thus, the reference is only an approximation and likely underestimates the real number of passengers.

Supplementary Note B: Symmetrized flows

We assume, that the observed system is in equilibrium, i.e. there is no population change due to the human mobility on the WAN. In other words, we neglect migration and assume that every visitor returns to its origin (5), i.e. the OD-flow T_A out of region A is the sum of the native population $\hat{N}_A$ and the visiting populations:

$$O_A = \hat{N}_A + \sum_{B \neq A} \hat{N}_B p_n(A|B) \tag{B}$$

with $p_n(A|B)$ as the import probability of only the native population. As a consequence, we expect the true OD-matrix to be symmetric, if it describes the human-mobility over a long time period. The shorter the time period represented by the OD-matrix, the higher it is influenced by fluctuations (e.g. not yet all visitors returned to their origin). Thus, we expect a larger asymmetry between distant countries (weakly connected countries), because the few visitors might stay longer.

We estimate the asymmetry by

$$a_{sym}(A,B) = \frac{|T_{AB} - T_{BA}|}{\max(T_{AB}, T_{BA})} \tag{C}$$

and observe for the reference OD-matrix the lowest asymmetry in mean and median compared to all others estimates (compare Fig. DM with A-L). The OD-matrices estimated by the import risk model (Fig. DD, H, L) are the most symmetric ones; however, the asymmetry is still twice as high than for the reference trip (compare Fig. DL with M). Additionally, the typical pattern of lower asymmetry for stronger connections is much less pronounced in the model estimates compared to the reference data.

We symmetrize the OD-matrix by (i) compute the estimated OD-matrix

$$T^{(0)}_{m,n} = p^{(0)}(m|n) N_n \tag{D}$$

from the import probability estimate, (ii) correct it by computing its symmetric part,

$$\mathbf{S} = (\mathbf{T} + \mathbf{T}^{\top})/2 \tag{E}$$

and (iii) compute the corresponding corrected import probability via

$$p^{(1)}(A|B) = \mathbf{S}_{AB}/S_B \ . \tag{F}$$

By going through these steps, import probabilities that represent a flow which is larger than the respective return flow are decreased and vice versa. However, the import probability after the first correction $p^{(1)}(A|B)$ results in a OD-matrix $\mathbf{T}^{(1)}$ of higher symmetry but with still a significant asymmetry, e.g. $p^{(1)}(A|B)$ for the gravity model with exponential distance decay and effective distance the median asymmetry decreases from $MED(a_{sym}) = 0.84$ to $MED(a_{sym}) = 0.15$. Thus, we recursively iterate $M = 3$ times through steps (i) till (iii), i.e. until $p^{(3)}(A|B)$, which returns for all models a comparable a_{sym} in mean and median to the reference data.

Alternative symmetrization. A possible reason for the asymmetry in the estimated ODs is that the import probability $p(A|B)$ only represent the import of the native population $p_n(A|B)$ from B but not the visitors from A returning from B. However, we are interested in the combined import probability $p_c(A|B)$ that we could in principle compute by

$$p_c(A|B) = \frac{T_{c,AB}}{T_{c,B}} \tag{G}$$

$$= \frac{T_{n,AB} + T_{n,BA}}{T_{c,B}} \tag{H}$$

$$= \frac{p_n(A|B)\hat{N}_B + T_{n,BA}}{T_{c,B}} \ . \tag{I}$$

The problem is, that we only know the WAN flow network $\mathbf{F}$ and thus the outflow per node

$$F_A = T_A + H_A \tag{J}$$

which is the OD-outflow and the transit passenger H_A through A. We could compute the combined import probability $p_c(A|B)$, if we assume that our estimated import probability is the one of the native population, i.e. $p_n(A|B)$, and if we could estimate the native populations $\hat{N}_B$. To do so, we assume that there are no transit passengers, i.e. setting in $H_A = 0$ in Eq. J we arrive at

$$\hat{N}_A = k_A F_A \quad \text{with} 0 < k_A < 1 \ . \tag{K}$$

Thus, it boils down to finding the coefficient vector **k** that estimates the combined OD-outflow

$$\tilde{T}_{c,B}(\mathbf{k}) = \sum_{A\neq B} \tilde{T}_{c,AB} \tag{L}$$

$$= \sum_{A\neq B} (\tilde{T}_{n,AB} + \tilde{T}_{n,BA}) \tag{M}$$

$$= \sum_{A\neq B} (p_n(A|B)\ k_B\ F_B + p_n(B|A)\ k_A\ F_A) \tag{N}$$

best compared to the true combined OD-outflow $T_{c,B} \approx F_B$. Note that we use $\tilde{x}$ to mark estimates based on **k**. It is a high dimensional optimization problem with a bounded parameter-search space. We used a simple square error function:

$$e(\tilde{\mathbf{T}}_c(\mathbf{k}), \mathbf{T}_c = \sum_A (\tilde{T}_{c,A}(\mathbf{k}) - T_{c,A})^2 \tag{O}$$

We tested available optimizers for a bounded search space from *scipy* (version=1.7.1, using 'scipy.optimize.minimize') by staying as close to the data as possible, i.e. using the observed outflow as native outflow and the estimated import probability as import probability of natives. The optimizer 'Powell' and 'trust-constr' performed well on various import probability estimates, while 'L-BFGS-B', 'Nelder-Mead', 'TNC' and 'SLSQP' did not converge to the correct solution. However, if applied to the real data, the optimizer fails for 8 of the 12 import probability estimates, i.e. the coefficients do not result in a symmetric OD-matrix (not shown). This suggests that the assumptions do not hold, i.e. the estimated import probability does not correspond to the import probability of natives and/or transit passengers cannot be neglected.

Supplementary Note C: On the overestimation of low import probabilities

The import risk model does overestimate low import probabilities (Figs. G), i.e.

$$p_\infty \propto \hat{p}^\alpha,\ \alpha \approx 2/3\ . \tag{P}$$

Here we present several attempts to understand mechanistically why this overestimation happens and introduce slight variations of the import risk model.

We study the influence of the flow scaling exponent ν that estimates the travelling population N_i of the airport i depending on its WAN outflow F_i via

$$N_i = F_i^\nu\ . \tag{Q}$$

The larger the exponent, the smaller the difference to the reference data (Fig. HA). That means if passengers are more likely to exit at larger airports, lower import probability are less strongly overestimated.

Instead, the effective distance offset d_0 does not change the overestimation at all (Fig. HB), which suggests that the differences in transition probability are too large to be influenced by penalizing hop distances.

Inspired by the fact that a larger exit at large airports decreases the overestimation and that large airports are rather at the beginning of the shortest path, we introduce a descendant fraction exit parameter μ that generalizes the shortest path exit probability from Eq. 6 to

$$q_i(n_0) = \frac{(1-\mu)N(i)}{(1-\mu)N(i) + \mu N(\Omega(i|n_0))}\ . \tag{R}$$

With $\mu = 0.5$, we recover Eq. 6 and with $\mu > 0.5$ we shift the exit to the descendant (or offspring) nodes. We verify the expected result, that an aversion of descendant exits decreases the overestimation of low import probabilities (Fig. HC).

Assuming that closer nodes on the shortest path tree are also geographically closer, we should observe a decrease in the overestimation if we weight the node populations with the inverse of their distance to the outbreak location (referred to in the main text as "geodesic distance weighted" exit probability). We find the expected relation (Fig. HD), i.e. the distance weighted import risk model overestimates less. Additionally, we find that overestimation further decreases if the effective distance is used for weighting.

Finally, we want to decrease the exit at nodes that have a large effective distance by setting the shortest path exit probability of the leaf- or dead-end nodes to a value smaller than the default value 1. The idea is that the random walker is not determined to end at leafs, but can walk on and is more likely return to hub nodes. Interestingly, the expected decrease in overestimation is only present for low leaf exit values as 0.1 (Fig. HE).

Disease/Variant	N	N_{C0}	N_{C1}	$N_{C0\ \&\ C1}$	$N_{C0\ \&\ C1\ \&\ C2}$
Theta	18	2	2	1	1
BA.3	29	2	11	2	1
Lambda	36	6	14	6	2
Zeta	37	4	20	3	3
Iota	43	7	11	7	5
Kappa	43	5	9	5	6
Epsilon	48	3	10	2	3
Mu	48	7	21	7	2
BA.2.75	54	5	24	5	1
Eta	72	13	40	12	2
Gamma	77	13	31	12	7
BA.5	88	30	53	30	24
BA.4	101	41	67	41	34
Beta	103	22	60	20	18
H1N1	115	54	53	38	38
BA.2	137	64	112	64	57
BA.1	143	65	115	65	48
Alpha	144	53	101	51	27
Omicron	158	98	145	97	67
Delta	159	68	131	65	62
COVID	231	134	164	131	115

Table A. Filtering criteria for the log-cases fit to extrapolate the arrival times t_A. A country is excluded if (C0:) the detection is too sparse before peak-0 (less than 6 weeks of data), (C1:) the number of cases at peak-0 is below 30 (otherwise the signal is too noisy), (C2:) the extrapolated arrival time is before the WHO-outbreak date. N is the number of countries for which case data could be generated. N_{C0} and N_{C1} are the countries that pass criteria C0 and C1. $N_{C0\ \&\ C1}$ and $N_{C0\ \&\ C1\ \&\ C2}$ are the numbers of countries that pass multiple criteria.

Supplementary Note D: Disease arrival time analysis

In the main text the disease arrival time t_A is estimated by the first reported case in a country. However, it is possible that the cases were tested (or sequenced in case of the SARS-CoV-2 variants) not regularly and the first case appeared actually before the first detection. This could be a source of noise and we use an alternative estimate of the first arrival time $\hat{t}_A$ which is the interception of a linear fit on the logarithmic number of new cases. The approach assumes that the disease/variant grows in the early phase exponentially. That is why we identify the first local maxima of new cases in each country, to which we refer to as *peak-0*, and use up to the 12 first data-points of its slope for the logarithmic fit (with the weekly resolution of the date sets, it corresponds to maximal the first 3 month). We exclude a country if (C0:) the detection is too sparse before peak-0 (less than 6 weeks of data), (C1:) the number of cases at peak-0 is below 30 (otherwise the signal is too noisy), (C2:) the extrapolated arrival time is after the WHO-outbreak date. The summary of the exclusion criteria is listed in Tab. A. For the arrival time correlation analysis we only included disease/variants with at least 10 countries that passed the criteria, leaving us with 10 diseases/variants. The case data of the variants V in a country is the number of new COVID-19 cases times $N_S(V)/N_S$, with $N_S(V)$ as the number of sequenced samples of the variant V and N_S the number of all sequenced samples.
We illustrated for the SARS-CoV-2 variant Alpha the new cases of the countries that passed all three criteria (Fig. Q) and 25 randomly selected examples for countries whose new case numbers did not pass (Fig. R). With the extrapolated arrival times t_A of the 10 diseases we reproduce the correlation analysis results of the main text (compare Fig. 6, P). The difference between the models becomes even smaller and some disease have a strongly decreased correlation. For example, for BA.2 and BA.1 the simple t_A estimation by first count correlation reaches values over 0.4 while the log-cases estimation for t_A stays below 0.2 for both.

References

1. Ettore Recchi, Emanuel Deutschmann, and Michele Vespe. Estimating Transnational Human Mobility on a Global Scale. *SSRN Electronic Journal*, 2019.
2. Ettore Recchi, Emanuel Deutschmann, and Michele Vespe. Global Transnational Mobility Dataset, 2019. https://doi.org/10.5281/zenodo.3911054.
3. Technavio. Dipping US Sales Are Forcing Online Travel Agencies to Look to Europe and Asia for Growth, 2015. https://blog.technavio.org/blog/dipping-us-sales-are-forcing-online-travel-agencies-to-look-to-europe-and-asia-for-growth.
4. Sean O'Neill. Lufthansa Now Drives More Than Half Its Bookings Directly, 2019. https://skift.com/2019/03/14/lufthansa-now-drives-more-than-half-its-bookings-directly/.
5. Vitaly Belik, Theo Geisel, and Dirk Brockmann. Natural Human Mobility Patterns and Spatial Spread of Infectious Diseases. *Physical Review X*, 1(1):011001, aug 2011.
6. World Bank. The World Bank: GDP per capita, 2023. https://data.worldbank.org/indicator/NY.GDP.PCAP.CD.
7. Technical Advisory Group on SARS-CoV-2 Virus Evolution. Historical working definitions and primary actions for SARS-CoV-2 variants, 2023. https://www.who.int/publications/m/item/historical-working-definitions-and-primary-actions-for-sars-cov-2-variants.

Disease/Variant	outbreak country	outbreak date	date of WAN
H1N1	Mexico (MX)	2009-02-01	2014
COVID	China (CN)	2019-12-01	2019
Beta	South Africa (ZA)	2020-05-09	2020-05
Alpha	United Kingdom (GB)	2020-09-05	2020-09
Epsilon	United States (US)	2020-03-14	2020-03
Eta	Nigeria (NG)	2020-11-07	2020-11
Delta	India (IN)	2020-10-03	2020-10
Omicron	South Africa (ZA)	2021-11-06	2021-11
Iota	United States (US)	2020-11-07	2020-11
Gamma	Brazil (BR)	2020-11-07	2020-11
Lambda	Peru (PE)	2020-12-05	2020-12
Kappa	India (IN)	2020-11-07	2020-11
Zeta	Brazil (BR)	2020-04-18	2020-04
Mu	Colombia (CO)	2021-01-09	2021-01
BA.1	South Africa (ZA)	2021-10-09	2021-10
BA.2	South Africa (ZA)	2021-11-06	2021-11
BA.2.75	India (IN)	2022-04-09	2022-04
BA.3	South Africa (ZA)	2021-11-20	2021-11
BA.4	South Africa (ZA)	2022-01-01	2022-01
BA.5	South Africa (ZA)	2022-02-05	2022-02

Table B. Disease and SARS-CoV-2 Variant outbreak information and WAN date. For each disease/variant the outbreak country and the date of the WAN used to compute the import probability estimates with the different models is displayed. Note that we only have the WAN from the years 2014 and 2019 in a yearly resolution and from 2020-2022 in monthly resolution. We repeated the analysis for COVID with the WAN from the month 2020-01-01, instead of using the yearly WAN from 2019, which gave comparable results.

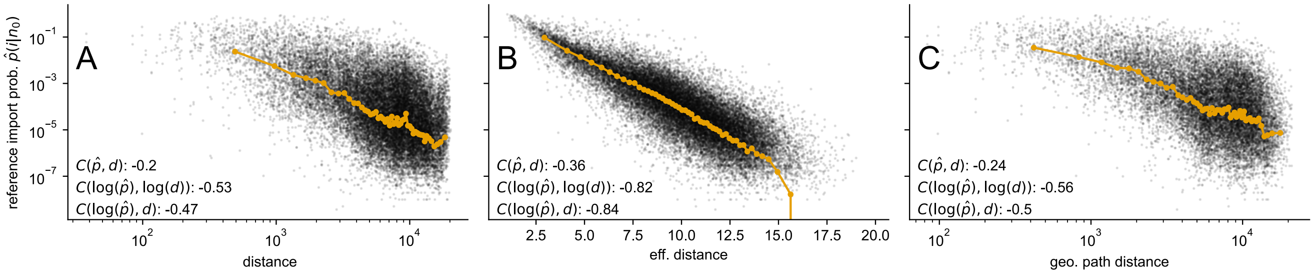

Fig A. Import probability dependence on the geographic distance (**A**), the effective distance (**B**) and the geographic path distance (**C**). The orange line represents the median and $C(x, y)$ is the correlation between the two measures either log-transformed or not. The geographic distance between countries is averaged over all airport pairs. The geographic path distance is the geographic distance along the shortest path derived from the WAN using d_{eff}, i.e. it is a combination of geographic and network information. The axis scale corresponds to the one with the highest correlation, i.e. log-log for distance and path distance (**A**, **C**) and y-log for the effective distance (**B**).

8. Kelsey Jordahl, Joris Van den Bossche, Martin Fleischmann, Jacob Wasserman, James McBride, Jeffrey Gerard, Jeff Tratner, Matthew Perry, Adrian Garcia Badaracco, Carson Farmer, Geir Arne Hjelle, Alan D. Snow, Micah Cochran, Sean Gillies, Lucas Culbertson, Matt Bartos, Nick Eubank, maxalbert, Aleksey Bilogur, Sergio Rey, Christopher Ren, Dani Arribas-Bel, Leah Wasser, Levi John Wolf, Martin Journois, Joshua Wilson, Adam Greenhall, Chris Holdgraf, Filipe, and François Leblanc. geopandas/geopandas: v0.8.1, July 2020.

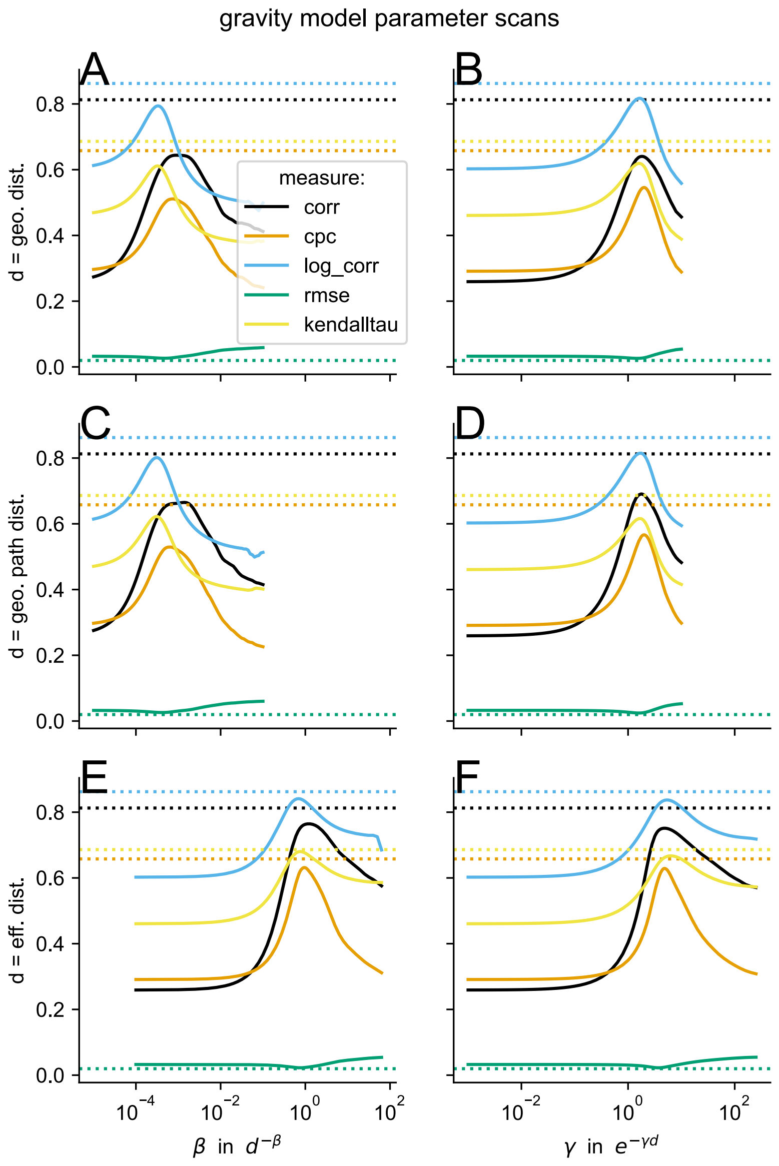

Fig B. Gravity model scans. Parameter dependence of measures that compare the model estimated import probability with the reference import risk $\hat{p}(i|n_0)$. Thereby is "corr" the correlation, "cpc" the common part of commuters, "log_corr" the correlation on log-scale, "rmse" the root mean squared error and "kendalltau" the rank correlation via Kendalls tau. Two versions of the gravity model are shown with an exponentially decaying distance function $f(d) = e^{-\gamma d}$ (left column: **A, C, E**), and a power law decaying distance function $f(d) = d^{-\beta}$ (right column: **B, D, F**). As distance the geodesic distance (first row: **A, B**), the geodesic path distance (second row: **C, D**) and the effective distance (third row: **E, F**) are used. The dotted horizontal lines show the comparison measure with the import risk as model and have the same respective color.

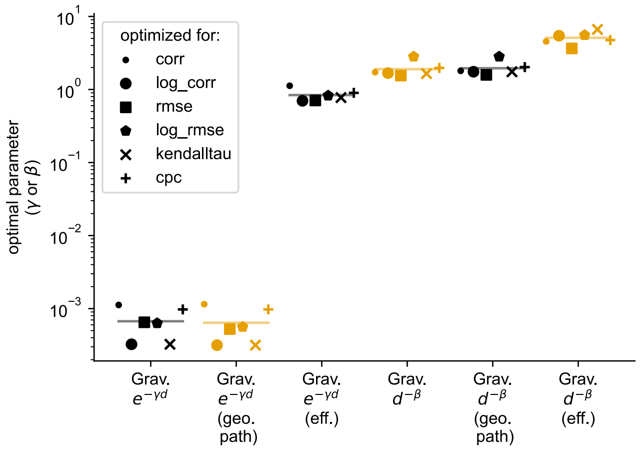

Fig C. Mean optimal parameters for gravity models. For each gravity model with exponentially and power law decaying distance function and with one of the three different distance measures (geodesic distance, geodesic path distance and effective distance), the exponent γ or β that results in the best fit to the reference import risk is shown. The comparison is quantified via the correlation (corr), correlation between the log-transformed import risks (log_corr), root mean square error (rmse), root mean square error of the log-transformed import risks (log_rmse), Kendall rank correlation (kendalltau) and the common part of commuters (cpc). The mean optimal parameter for each model is marked by a horizontal line and their values are $\gamma = [6.71, 6.41] * 10^{-4}$ for geographic and geo. path distance and $\gamma = 0.84$ for the effective distance, and $\beta = [1.90, 1, 95, 5.10]$ for geo., geo. path, and effective distance, respectively.

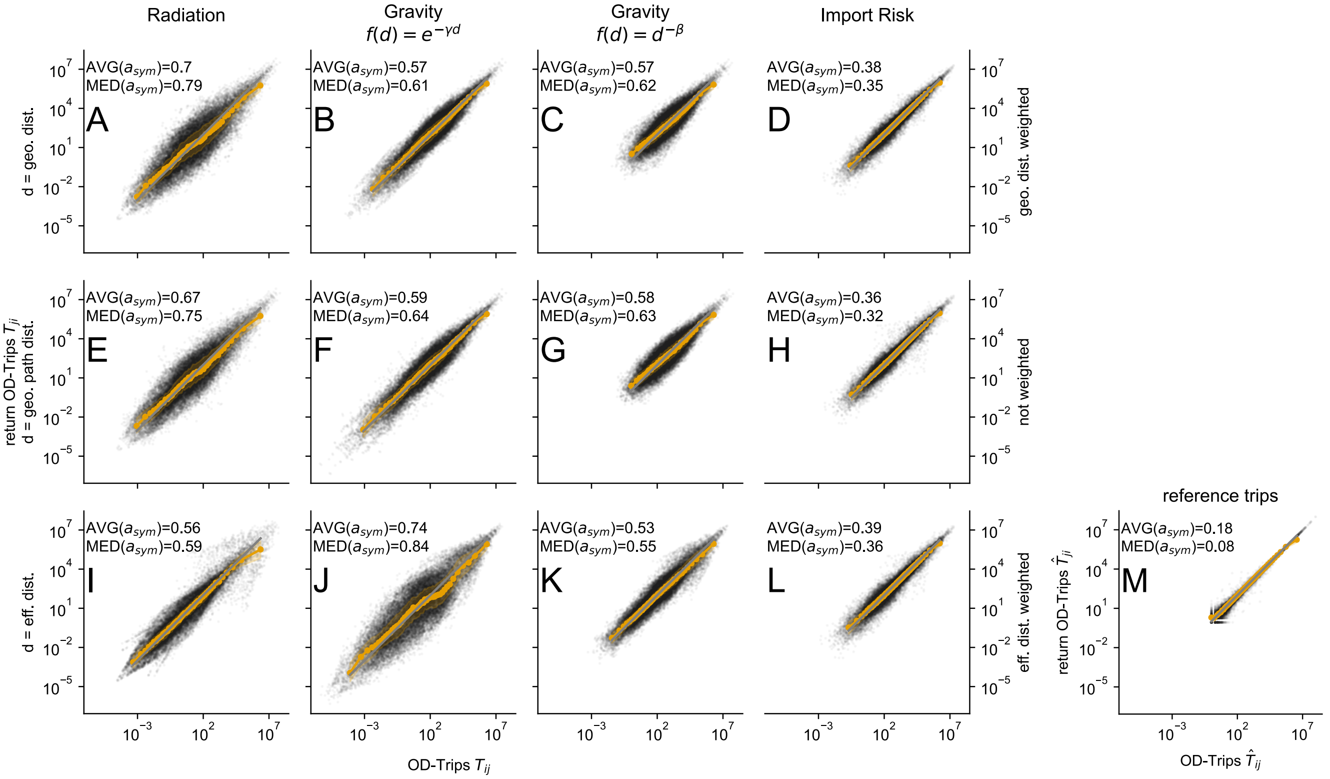

Fig D. Symmetry check for OD-matrix. Each dot represents the number of passengers that travel between 2 countries and back. The OD-matrix is computed by the radiation model (1st. column), gravity model with exponentially (2nd column) and power law decaying (3rd column) distance function and by the import risk model (4th column). The OD-matrix of the models is computed by multiplying the import probability with the source-outflow. The reference trips and return trips have the highest symmetry (5th column, **M**). The orange line depicts the median and the gray line is $y = x$ and illustrates perfect symmetry. The mean (AVG(a_{sym})) and median (MED(a_{sym})) asymmetry of the flows, computed according to Eq. C, are shown in each panel. The reference trips (**M**) show the lowest asymmetry, especially for large passenger flows.

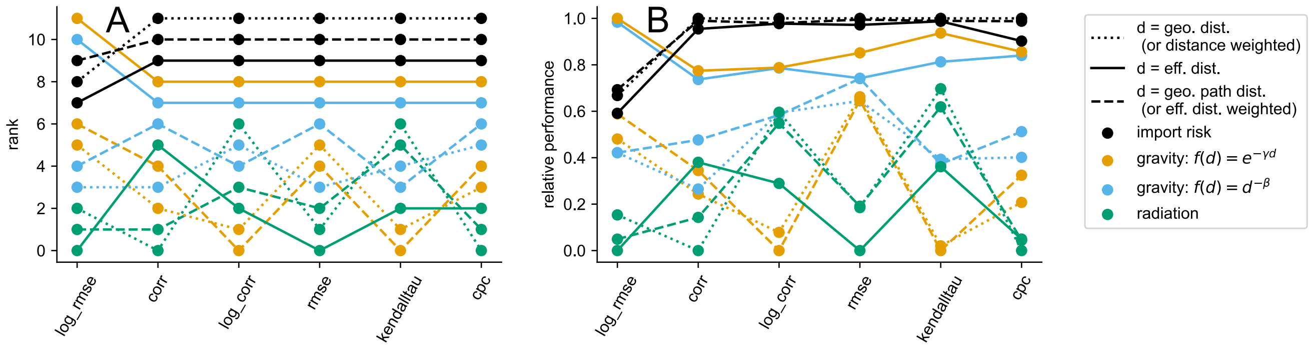

Fig E. Relative comparison measures for the import probability estimates. The rank (**A**) and the relative performance (**B**) for the different import probability estimation models. The model that agrees best (worst) with the reference import risk according a specific measure has the highest (lowest) rank and a relative performance of one (zero). The relative performance is then a linear interpolation between the best and worst model. The comparison measures are the correlation (corr), correlation between the log-transformed import risks (log_corr), Root-mean-square error (rmse), Root-mean-square error of the log-transformed import risks (log_rmse), Kendall rank correlation (kendalltau) and the common part of commuters (cpc). As exponents of the gravity models the mean optimal parameter is used (horizontal lines in Fig. C).

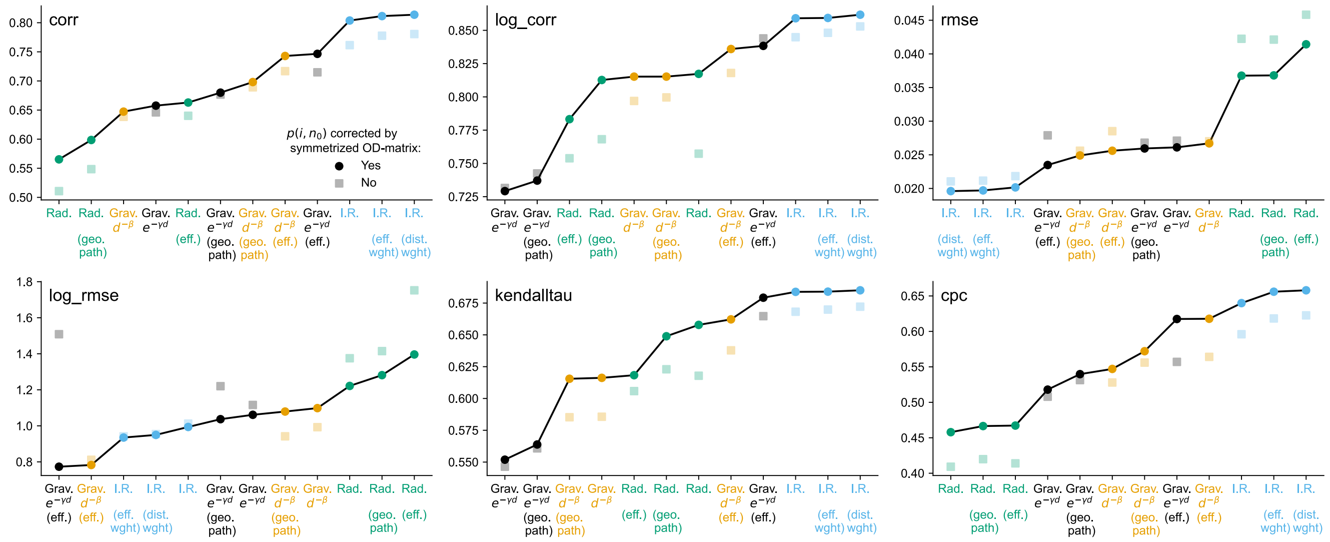

Fig F. Absolute comparison measures for the import probability estimates. The comparison measures are the correlation (corr), correlation between the log-transformed import risks (log_corr), Root-mean-square error (rmse), Root-mean-square error of the log-transformed import risks (log_rmse), Kendall rank correlation (kendalltau) and the common part of commuters (cpc). As exponents of the gravity models the mean optimal parameter is used (horizontal lines in Fig. C). The colors depict the 4 different models. The solid circles are the models with corrected import probability by symmetrizing their OD-matrix, and the transparent squares are the non-corrected import probabilities of the respective model.

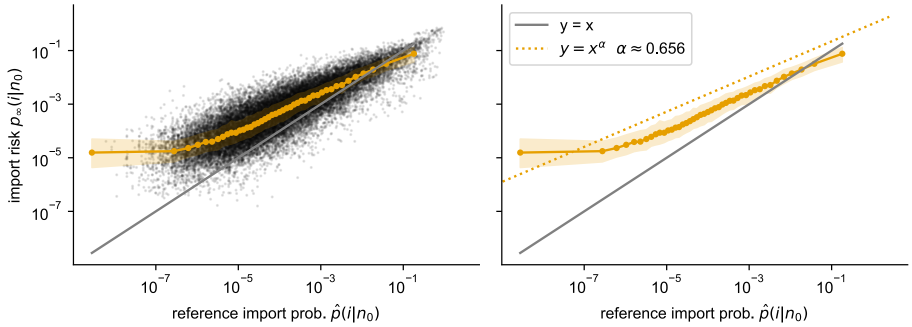

Fig G. Import risk comparison and its deviation from a linear relation. Scatter plot (left) and only median and IQR with an exponential fit (right).

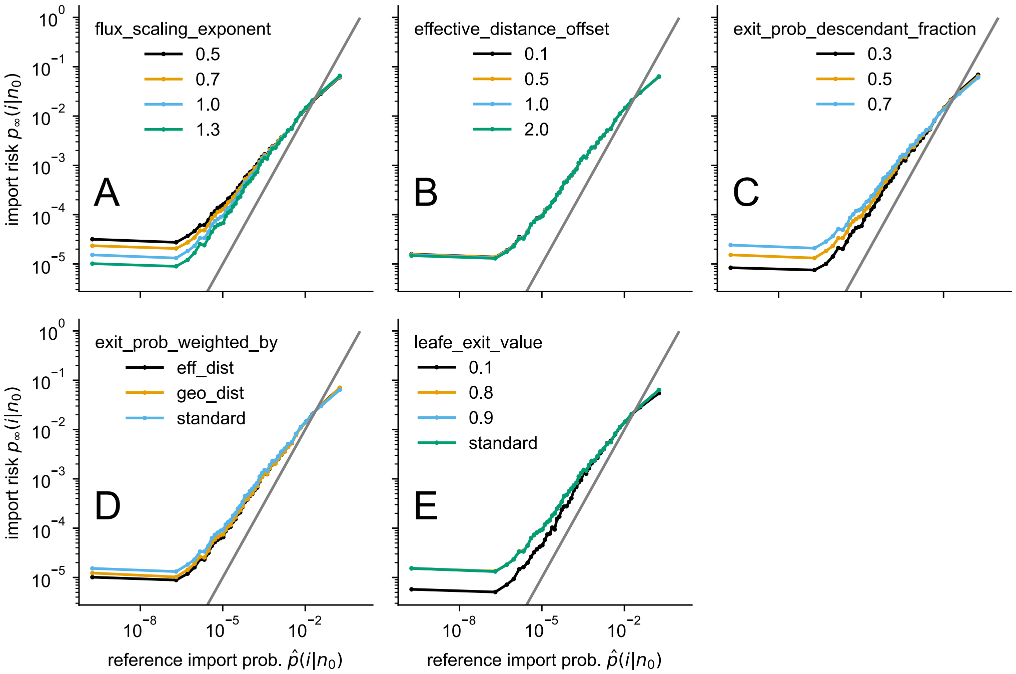

Fig H. Variations of the import risk model to investigate how additional parameters influence the relation between the import risk and the reference import risk. **A**: the flow scaling exponent ν that estimates the travelling population $N(i)$ of the airport i depending on its WAN outflow F_i via $N(i) = F_i^\nu$ (default: $\nu = 1$). **B**: the effective distance offset d_0 that penalizes larger hop-distances in the effective distance $d_{\text{eff}}(i|n_0) = d_0 - \ln(P_{ij})$ when creating the shortest path tree (default: $d_0 = 1$). **C**: the descendant fraction introduced in the shortest path exit probability, where 0.5 is the default value and values larger than 0.5 mean that the exiting at the descendant (or offspring) nodes compared to the current node becomes more likely. **D**: different weight options introduced for the shortest path tree exit probability. Per default, the node populations are not weighted. The weight is the inverse of either the geodesic or the effective distance. **E**: manually set shortest path exit probability of leaf nodes (dead-end nodes). Per default, the exit probability is 1. A decrease to 0.9 or 0.8 does not visually change the median.

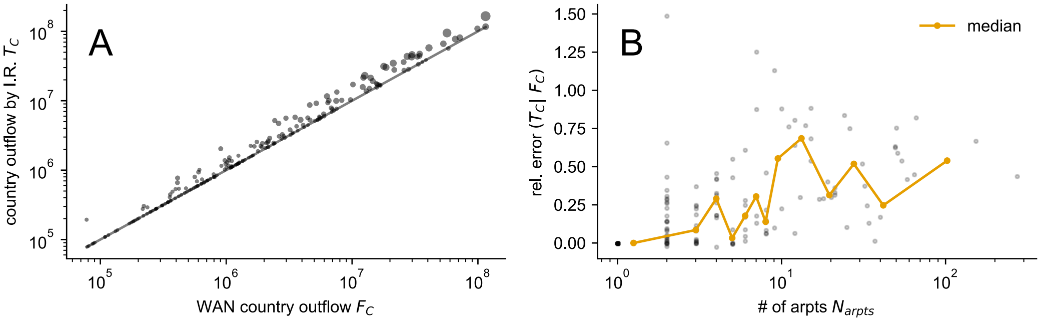

Fig I. Country outflow reconstruction by import risk. The flow in the WAN leaving a country F_C is estimated by the import risk model by $T_C = \sum_{n \in C} \sum_{m \notin C} p_\infty(m|n) N_n$. Both measures are directly compared (**A**) and the relative error is computed depending on the number of airports in the respective country N_{arpts} (**B**). The import risk model does not include the concept of a country which partly explains the overestimation for larger airports. Another explanation is the overestimation of the respective airport population $N_n = F_n$ by the WAN outflow for the import risk model (the true population is smaller because of the transit passengers that need to be excluded). Note that the WAN is used here, i.e. we check for self-consistency of the model and no reference data is included.

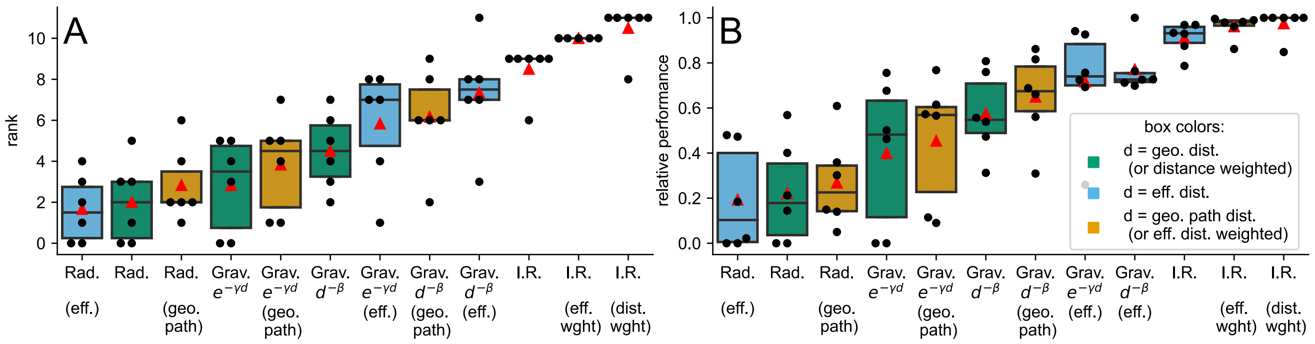

Fig J. Uncorrected models: rank and relative performance. Same analysis as in the main text in Fig. 4), however, here the uncorrected model predictions are used, i.e. without symmetrizing the OD-matrix.

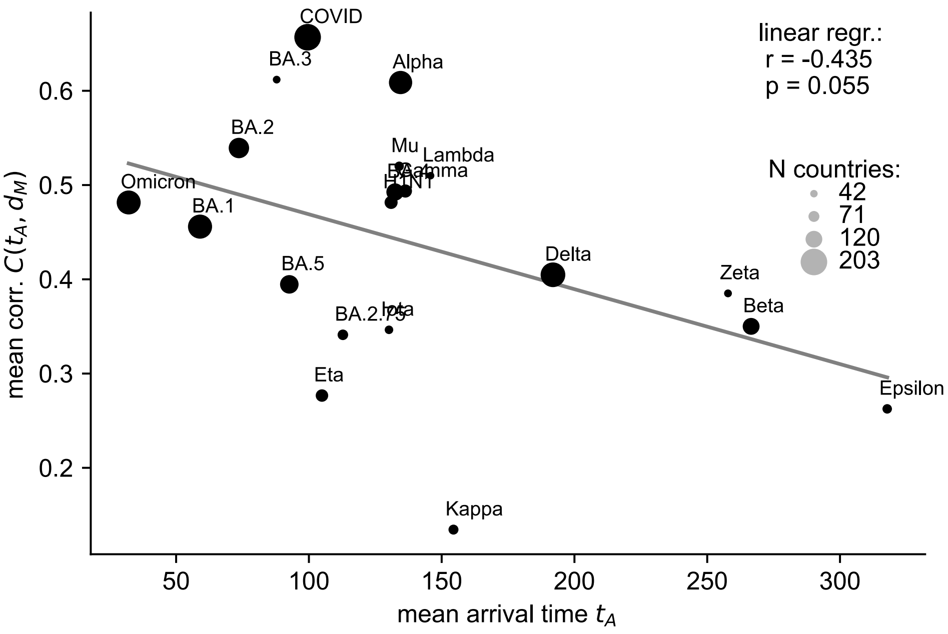

Fig K. Mean correlation between arrival time and effective model distance vs. the speed of the disease estimated by the mean arrival time $\langle t_A(C)\rangle_C$, averaged over all countries C. The correlation $C(t_A, d_M)$ between arrival time t_A and effective model distance d_M is averaged over all models. The size of the datapoints illustrates the number of countries that were reached by the disease.

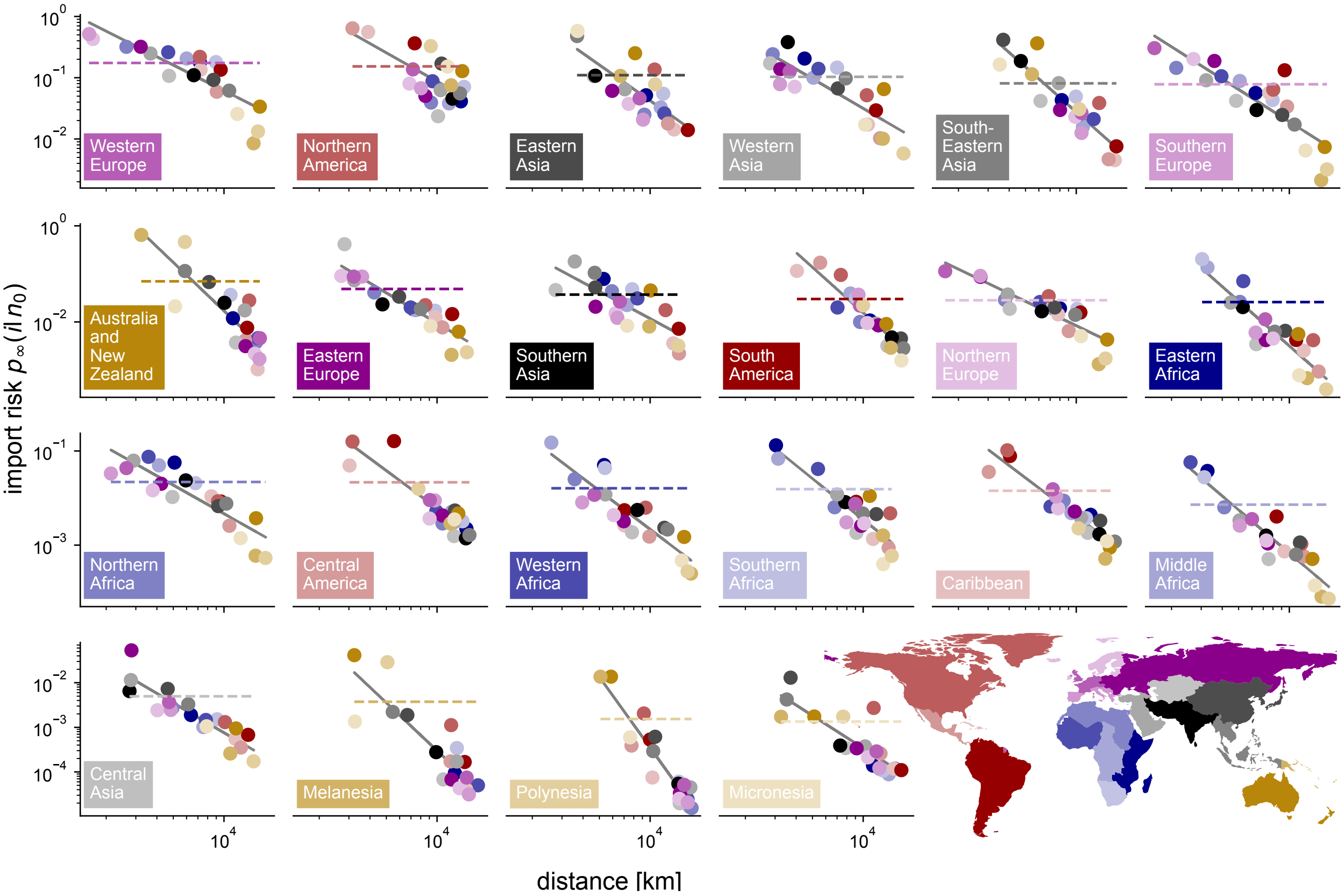

Fig L. Import risk between world regions to a specific target region. In contrast to its derivation the import risk is displayed in a target-centric view, i.e. each panel displays the import probability to a single target region from all source regions. The distance between world regions is the mean distance between their airport locations. The grey line represents a power-law fit $p_\infty = c \cdot d^{-\alpha}$. The mean import risk is marked for each world region by a horizontal dashed line. The 22 target-world-regions are sorted according to their mean import risk. Maps are created with geopandas (8).

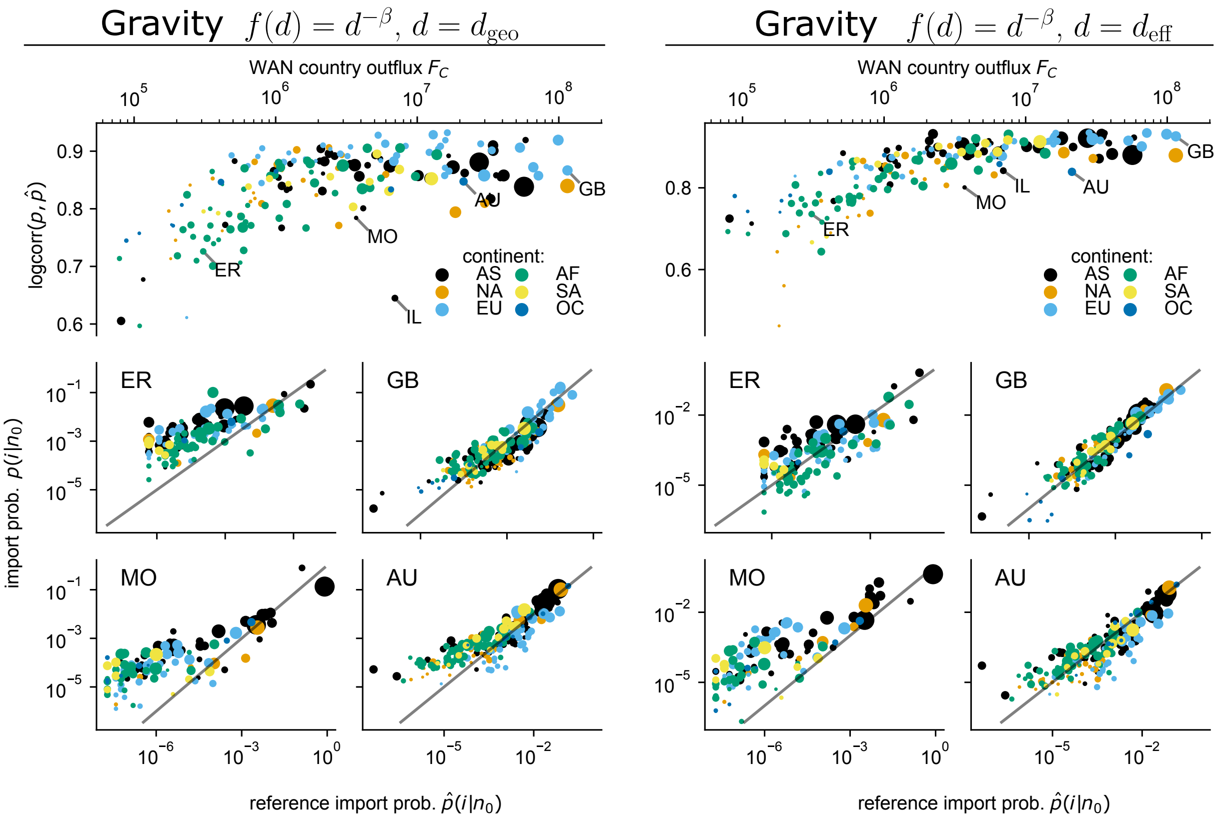

Fig M. Source countries prediction quality and WAN outflow for two gravity models. Same model-result representation as in Fig. 7 but here instead of the import risk model, the gravity model with power-law distance decaying function using the geodesic d_{geo} (left) or effective d_{eff} (right) distance is applied. Also for these models the logcorr between import probability estimates $p(i|n_0)$ and the reference data $\hat{p}(i|n_0)$ improves for countries with a larger outflow in the WAN.

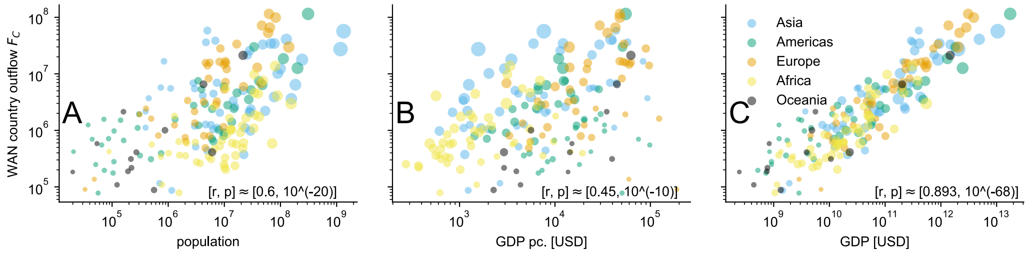

Fig N. WAN flow out of countries vs. population and GDP The WAN flow out of a country is best mapped by its gross domestic product (GDP, **C**) compared to its population (**A**) or per capita GDP (**B**). The linear double-logarithmic regression results are shown in the lower part of each panel (r- and p-value). The size of each country corresponds to its population (**A**) and the color codes its continent. GDP is taken from the World Bank Dataset for the year 2014 (6).

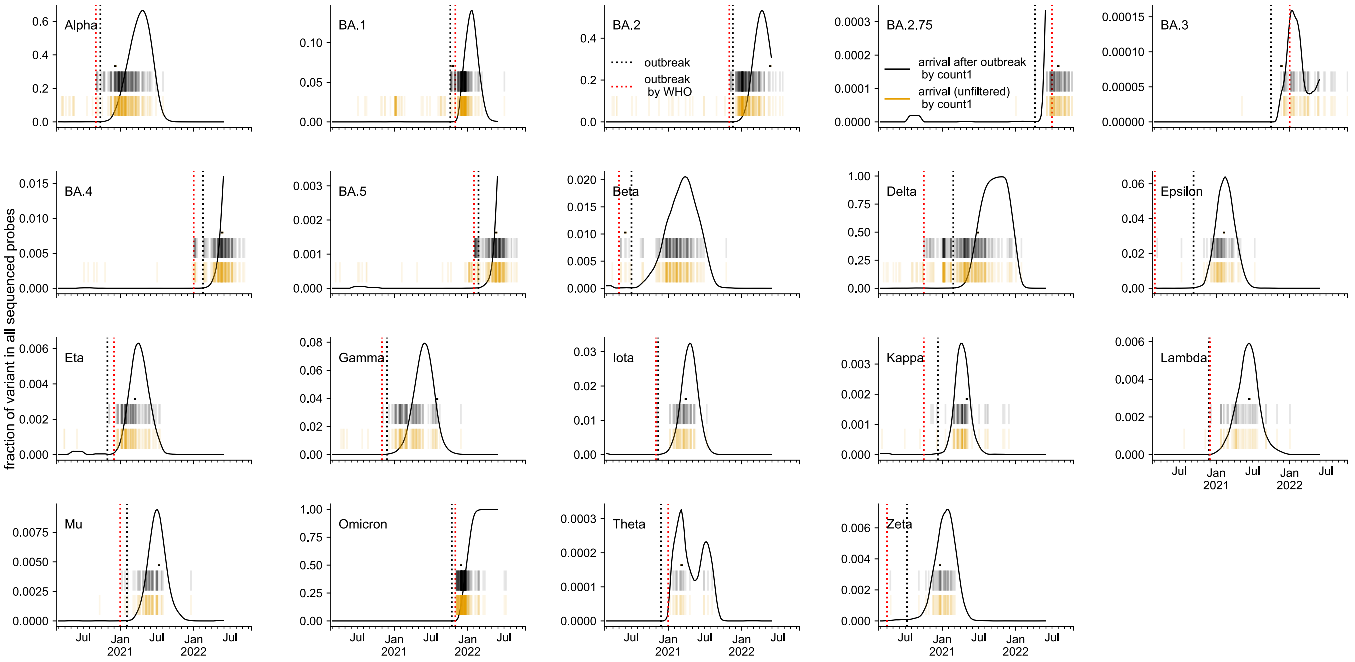

Fig O. Variant outbreak detection and fraction of sequenced samples for each of the considered variants. To illustrate the spread of the variant and how often it occurs worlwide the fraction of the variant in all sequenced probes is plotted, i.e. if it reaches 1, all sequenced probes are the respective variant. The official WHO outbreak date (7) is highlighted as red dotted vertical line. We estimated an outbreak date by 45 days before the fraction of sequenced samples reached 2.5% of its world-wide peak. The orange vertical lines (lower row of lines) show for each country the arrival of the variant, estimated by the first sequenced probe ("count1"). The black vertical lines (upper row of lines) show the arrival times after the outbreak which are used in the main text.

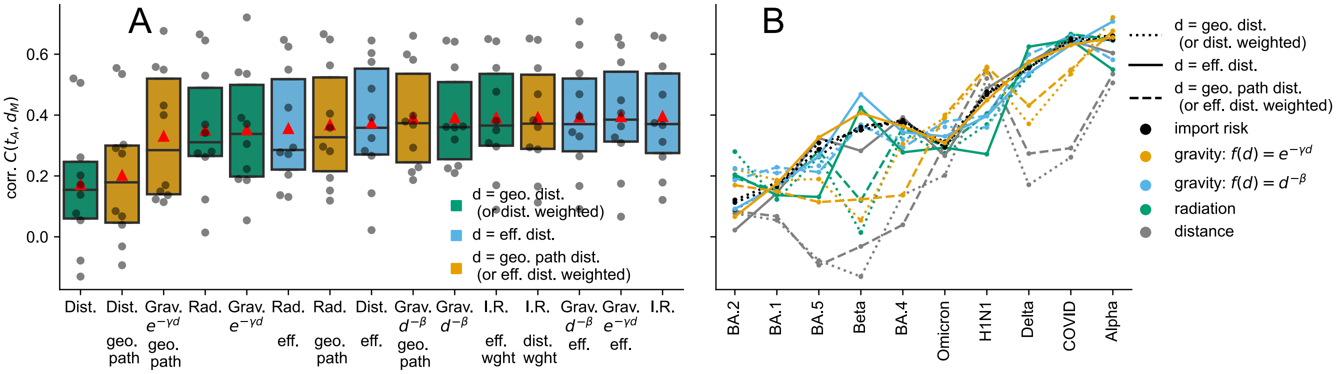

Fig P. Correlation analysis with log-cases estimated arrival time. Each model's import probability is converted to an effective distance $d_M(i|n_0) = -\ln(p(i|n_0))$ with n_0 as the outbreak country of the respective disease. The correlation results $C(t_A, d_M)$ with the arrival time $t_A(i)$ of the disease in the target country i are grouped by model (**A**) and by the disease (**B**). As comparison distances, the correlation of the geodesic, geodesic path (on the effective shortest path tree) and the effective distance with t_A are shown. Each dot represents a correlation result of the 10 considered outbreaks (H1N1 in 2009, COVID-19 in 2020 and the spread of 8 of its variants in the years 2020-2022). For the analysis only those diseases/variants were used with more than 10 datapoints (see Tab. A).

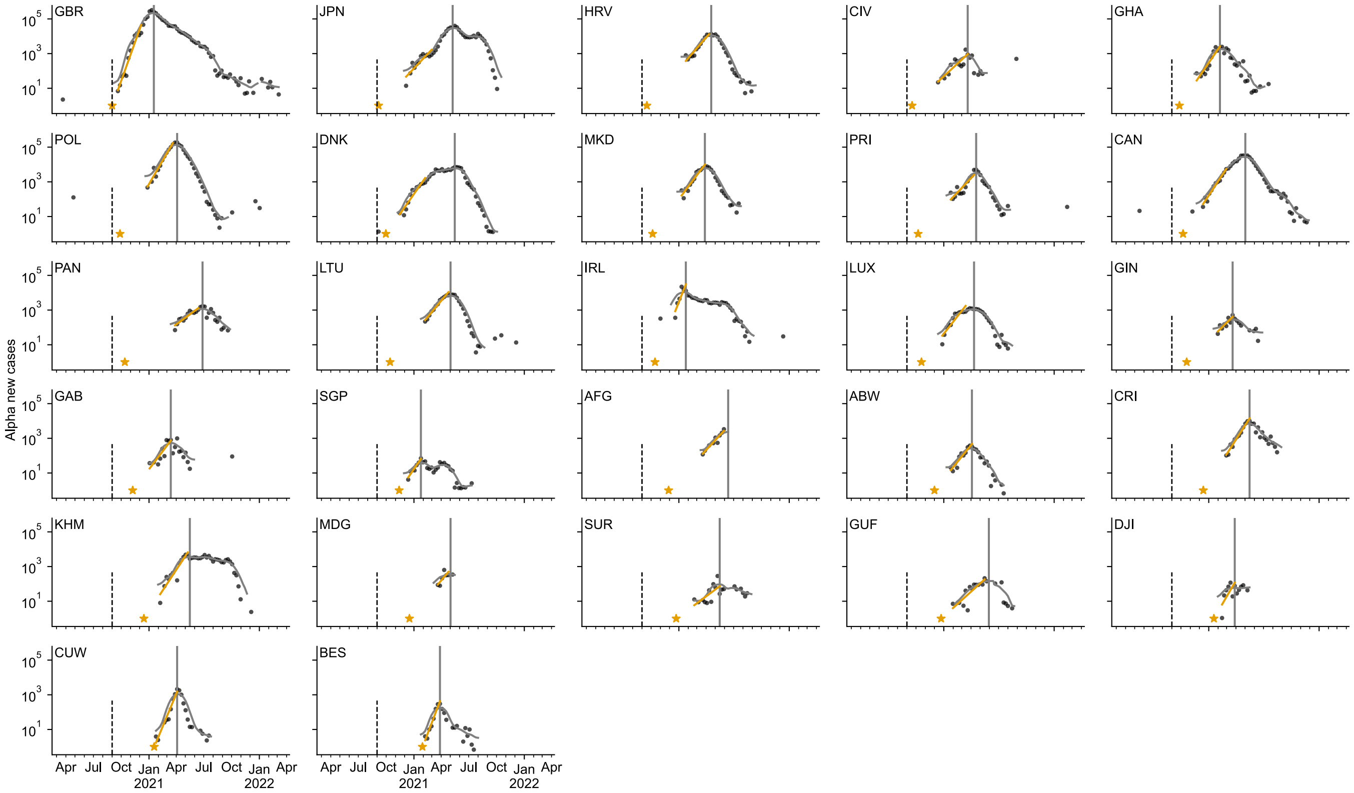

Fig Q. New case numbers of the Alpha variant for countries that passed the selection criteria for the log-cases fit to extrapolate the arrival time t_A in the attempt to reduce noise. The vertical dashed line marks the outbreak as listed by the WHO (7), the yellow star is the extrapolated arrival time from the log-cases fit that is illustrated by a yellow line. To determine the peak-0 (marked by a vertical line) we used a difference analysis on the smoothed new-cases data.

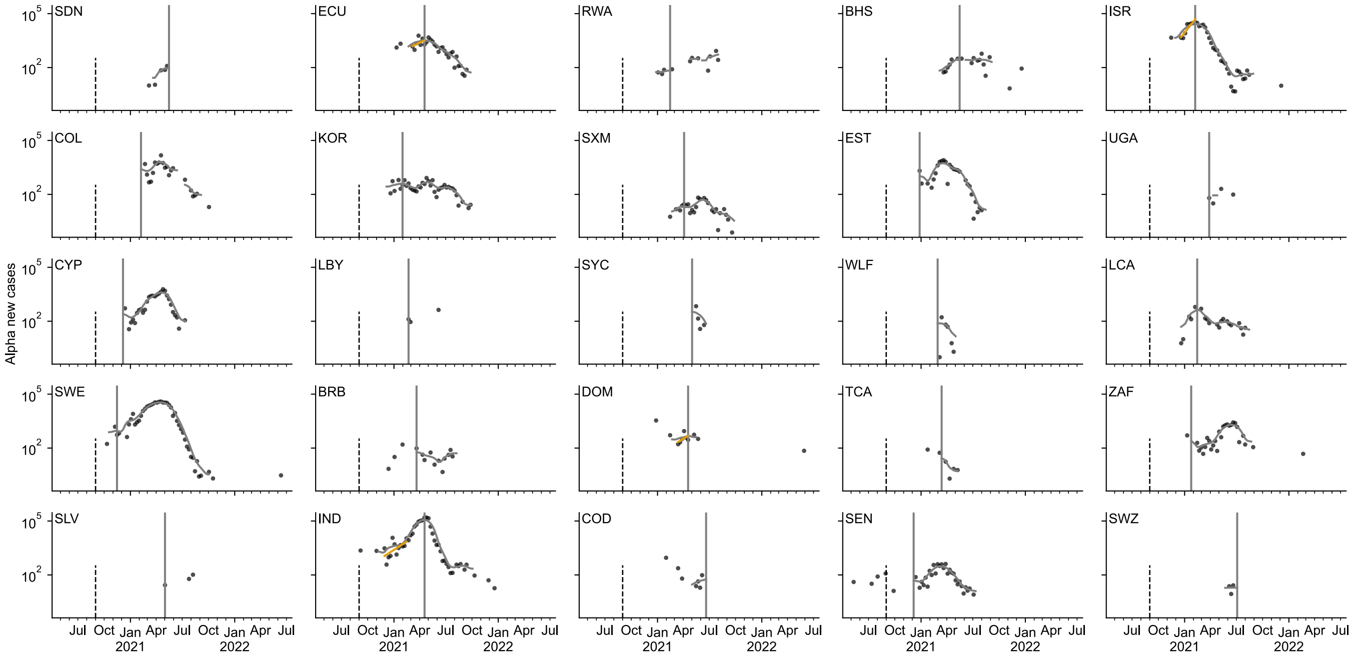

Fig R. New case numbers of the Alpha variant for countries that failed the selection criteria for the log-cases fit to extrapolate the arrival time t_A in the attempt to reduce noise. The vertical dashed line marks the outbreak as listed by the WHO (7). Those countries that passed the criteria C0 and C1 (see Tab. A for details) show the log-cases fit. Note that the latter have an extrapolated t_A before the outbreak date listed by the WHO. To determine the peak-0 (marked by a vertical line) we used a difference analysis on the smoothed new-cases data.
