# Supplementary material for: Inferring country-specific import risk of diseases from the world air transportation network
Source: PLoS Comput Biol. 2024 Jan 24;20(1):e1011775. doi: 10.1371/journal.pcbi.1011775 (PMC10843136; doi:10.1371/journal.pcbi.1011775)
Supplement: Attachment — Submitted filename: ResponseLetter.pdf [file pcbi.1011775.s003.pdf]

HUMBOLDT-UNIVERSITÄT ZU BERLIN

HU | Faculty of Life Sciences | Institute of Biology |
10115 Berlin | GERMANY

Faculty of Life Sciences

Institute of Biology

Dr. Pascal P. Klamser
Modelling of
infectious disease and mobility

Address:
Humboldt-Universität zu Berlin
Invalidenstr. 42
10115 Berlin
GERMANY

Phone:
+49 30 187542070
+49 1573 0063961

klamser@physik.hu-berlin.de
www.hu-berlin.de

Office Location:
Nordufer 20
13353 Berlin
GERMANY

Prof. Yamir Moreno
& Dr. Thomas Leitner
PLOS Computational Biology

Datum:
26. Sept. 2023

Dear Prof. Yamir Moreno,
Dear Dr. Thomas Leitner,

we thank you very much for considering our manuscript for PLOS Computational Biology. We have addressed the comments of the reviewers in detail, to whom we are very thankful for their time and energy. Below, we listed a summarization of the manuscript adaption motivated by the main concerns of both reviewers.

We have examined the text for grammatical errors, firstly with the help of Reviewer #1's comments and secondly with an independent correction of the entire text. Many small changes in the text are due to this. We have also critically evaluated Reviewer #1's comment on symmetrization and incorporated his concerns about this into the discussion. However, we believe that the current manuscript had already analyzed this step in sufficient detail. Nonetheless, the adapted discussion makes reviewer #1's point clear and we believe it is an important contribution, since it pushes the point, that our model might be prone to work better for high income country than low income countries.

Both reviewers had expressed concerns that the manuscript had too weak a reference to disease spread. We agree with the reviewers and have supplemented the manuscript with an analysis on the arrival times of 20 diseases or SARS-CoV-2 variants. The new analysis shows that the models using the effective distance as a measure perform comparably well among each other. We also suggest that the available data are probably not sufficient to reproduce the advantage of the import risk model, as the differences obtained with the logarithmic correlation between the import probabilities and the reference probability are too small at 7%. We have tried to reduce the error in the data and thus increase the data quality by estimating the arrival time of the diseases more accurately with a fit of the logarithmic case numbers. However, the number of cases or sequencing rates were too low in many countries to use this method, so many arrival times had to be excluded and in the end many fewer countries remained for analysis. We have summarized this part in the Supplementary Information with additional Figures and tables.

We have addressed in detail reviewer #2's concern that we have not sufficiently represented existing models that calculate the import probability of disease. We have incorporated the suggested sources in the manuscript and hopefully made it clear that it was not intentional, but rather that we focused

on the mobility models that are also used in the model suggested by reviewer#2.

Reviewer #2 had asked us to better illuminate our results regarding the poor performance of the radiation model. We have explained in the section introducing the alternative models why we believe that the radiation model should perform well in the case of the WAN. In the discussion, we shed light on why it performs poorly despite the new distance measure we used in the model and fully agree with reviewer #2 on the main points. Nevertheless, we think that the radiation model has a justification, firstly because it is well established in the human mobility community and secondly because it is a paremeter-free model and we need exactly such a model for our comparison with our parameter-free model.

We hope you find the updated manuscript a better fit for publication and thank you for your consideration.

Yours sincerely,

Pascal Klamser
(on behalf of the authors)

Response to the reviewers:

Reviewer #1: Inferring country-specific import risk of diseases from the world air transportation network

The authors propose an “import risk” model, based on an import probability that is determined by the effective distance (according to which the higher the passenger flow in air transportation between two points, the closer the distance) and the (a) shortest possible path between two points. The length of the paths is in turn defined by means of a random walk on a tree. Theoretically, the model is very interesting, but its applicability is blurred because determining the effective distance in real life is extremely difficult due to the lack of a database that serves as a baseline.

I only have two major comments:

There is a problem with English grammar that makes the article difficult to read. I pointed to several instances in my minor comments but doubtlessly there are many more to be corrected. The article needs to go through a thorough grammar revision before acceptance.

We are very grateful to all your suggestions and implemented all and checked the text for similar grammar problems. Additionally, we read the text and used a grammar checking tool (LanguageTool) to spot additional mistakes and gave the whole text a thorough reevaluation regarding the writing style. It improved the readability greatly and therefore the quality of the manuscript. Again, thanks!

The import risk model is very interesting mathematically. Nonetheless, the connection to import disease is loose. That is, despite suggesting the connection in the title and the abstract, the paper does little to justify this connection and exploit it. I think the paper is better fitted for PLOS One than for PLOS Computational Biology.

We concur with the referee's note that the initial manuscript had a too loose connection to real-world epidemics. This was primarily because the manuscript served as a model description meant to complement another paper. The latter paper utilized the import risk model within a global epidemic model to estimate the pandemic potential of SARS-CoV-2 variants using data that was both limited and noisy (Klamser and d’Andrea et al., "Enhancing global preparedness during an ongoing pandemic from partial and noisy data," PNAS Nexus 2023). Our intention was to maintain a clear separation between the results. However, we realized, triggered by the input of you and referee #2, that in the context of model comparison, there was essentially no overlap in the analysis. Thus, we performed an arrival time analysis of past pandemics. We computed the correlation between the negative logarithm of the import probability estimates and the arrival times of several significant outbreaks. These outbreaks included the 2009 H1N1

outbreak, the 2019 COVID-19 outbreak, and the spread of 18 SARS-CoV-2 variants. The variants were selected based on their classification as variants of concern or variants of interest and if there is sufficient data available (spread across multiple countries).

We added the section “Model comparison: Disease arrival times” with a new Figure (Fig. 6), a new paragraph in the discussion (5th paragraph), a part in the Methods, several figures and Tables in the SI and a part in the SI where we explore an alternative estimation of the arrival time via a log-cases fit (to reduce noise).
We observe that the models employing the concept of effective distance reach similar correlation results and outperform the others. That means, as long as the WAN information is considered, the course of a pandemic is represented realistically. We highlight in the discussion that the data is too sparse to recover the differences between import probability estimation models that we detected in comparison to the reference import probability data.

We believe that the new version of the manuscript is now better fit to the scope of PLOS Computational Biology, that has in fact numerous excellent publications that deal with human mobility in the context of epidemics.

A more general comment: The symmetrization of the OD matrix is a very strong assumption that can lead to an overestimation of the import risk for countries with high levels of migration. For instance, it is not common for people from first-world countries to migrate to third-world countries, whereas the opposite is much more common, and this proportion does not seem negligible. Therefore, the import/export ratio is less than 1.

We agree with the referee that the symmetrization of the OD matrix could be problematic, if migration is not negligible. However, we have shown in Figure S4 that the symmetry in the reference OD matrix is much higher compared to the other datasets. With the proposed symmetrization method, we arrive at comparable symmetry values for all models under consideration. We have also ensured that our results are robust and do not depend on the symmetrization as discussed in the 3rd paragraph of the “Discussion and Conclusions” section. There we refer to Fig. S6, where one can observe that the import risk method is still outperforming the other measures if no OD-symmetrization is added. Therefore, we believe to have treated the symmetrization with enough care and critical analysis. However, the aforementioned methodology does not invalidate your arguments and we believe them to be important as well (underrepresentation of low-income countries is already strong in the WAN and our analysis and proposed methods should not intensify it). Instead of running another additional analysis, we now end the 3rd paragraph of the discussion with: “It's crucial to note that the assumption of returning visitors is applicable when visitors and tourists dominate while migrants can be disregarded. However, this assumption may not hold for links between low- and high-income countries or conflict regions.”

Minor comments:

Page 1, column 1: “as shown for the distribution of over 400 invasive species” “as shown by the distribution of over 400 invasive species.”

Thank you, we corrected it.

Page 1, column 2: “Already the first plague pandemic that started 541 in the Nile” Is this B.C. or A.D.?

It is AD. Thanks for pointing it out, we corrected it.

Page 1, column 2: “According to the effective distance, region B is closer to region A if the passenger flow from A to B is larger than to other destinations”. This clearly needs a third region C to make the comparison meaningful: B is closer to region A than to region C if the passenger flow from A to B is larger than that from B to C.

Right, the current form was incomplete. However, we try to keep it as simple as possible, and want to avoid the naming of a region if it is not necessary. We modified the text, so it works only with A and B by using the extreme form: “According to the effective distance, region B is closest to region A, if the passenger flow from A to B is greater than to other destinations.”

Page 1, column 2: “… from a specific source to target” “from a specific source to a target”.

Thank you, we corrected it.

Page 2, column 1: “That means to exactly compute…” “This means that to exactly compute…”

Thank you, we corrected it.

Page 2, column 1: “However, none of the above approaches use the effective distance with its validated link to disease propagation and none is based on a mechanistic distribution”. First, how is the link of effective distance to disease propagation “validated”? Because of the qualitative connection in the second paragraph? It sounds like this point needs at least some explanation and additional references. Second, what does “mechanistic distribution” mean? “Mechanistic” is again used in the next sentence, but its meaning is still unclear.

We highly value this comment on the perception of the text. With validated in the context of diseases propagation, we indeed mean the qualitative connection between the effective distance and the arrival times of diseases from the second paragraph. We changed “validated” to “qualitative”, since qualitative is more precise than validated. We also thank the reviewer for pointing to the missing clarification of “mechanistic”. We added the sentence: “To our understanding, a mechanistic process mimics the detailed movement behavior of the passengers on the traffic network, and neither uses only quantities of and between the locations (gravity and radiation model) nor relies on principles of system in thermodynamic equilibrium (maximum entropy model), in other words it is a bottom-up approach.” We keep the explanation general, since we have not introduced the import risk model at this point of the text. But to be clear, the import risk model is based on probabilistic decisions at every airport, where the probabilities reflect the traffic outflow from the airport where the random walker is currently at.

Page 2, column 1: “We find that the import risk model outperforms alternatives and only marginally improves if geodesic distance information is included”. Rewrite this sentence.

We are not sure how to make the point more clear and not directly see a problem with the sentence structure. We rephrased it slightly: “We find that the import risk model outperforms the alternative models and improves only slightly when it includes not only WAN information but also the geodetic distance between airports.” and hope it is more clear now.

Page 2, column 2: Eq. (1) defines effective distance, but the definition of d_0 is extremely vague. From the previous explanation, it seems that it depends on i and j (the connecting flights between the two), so it is not constant. It is better to define it explicitly to avoid confusion.

We agree that the initial version was confusing, since Eq. (1) is the definition for a direct flight connection. We changed the sentence before Eq. (1) from “Additionally, it increases the distance by a constant d_0 for every connecting flight:” → “Together with a constant distance offset d_0, the effective distance between directly connected airports is”. The sentence that follows Eq. (1) specifies the additive nature of the effective distance for connecting flights. Following this sentence we added a short explanation on the effect of d_0: “Note that a distance offset of $d_0=0$ would make two routes indistinguishable as long as the product of the transition probabilities along each route is the same, but with $d_0>0$ the one route with fewer connecting flights is effectively shorter. ”

Page 2, column 2: “(i) a random walk with exit probability”. Exit probability of/from/to…? Even though the concept is formally defined below, this heuristic must be clearer.

Thank you for this comment. This part was definitely too short and we extended it. Now we are more specific in the exit probability and also mention that it is the connection element between the random walk and a the distribution mechanism, that is explained further below and was therefore to connect with. The current form of the text reads:

“The idea behind the import risk model is a combination of (i) a random walk with exit probability and (ii) a distribution mechanism derived from the $\deff$ SPT (Fig. 2). Applying a random walk is motivated by Iannelli et al. (17) who could improve the arrival-order prediction of $\deff$ by including all possible paths.”
→
“The idea behind the import risk model is a combination of two elements: (i) a random walk with an exit probability of the walker to finish its travel at the current node and (ii) a distribution mechanism derived from the $\deff$ SPT (Fig. 2). The use of a random walk is motivated by Iannelli et al. (17) who could improve the arrival-order prediction of $\deff$ by including all possible paths. The exit probability enables us to combine the random walk with a distribution mechanism that assigns the likelihood of each node being the final destination, as explained in detail in the second step.”

Page 2, column 2: “Applying a random walk is motivated by…” “The use of a random walk is motivated by…”.

Thanks, your proposal reads much better.

Page 3, column 1 (before eq(4)): “… all paths of length l to” “all paths of length l as”.

Thanks, we corrected it.

Page 3, column 2: Eqs. (5) and (6), the core of the paper, are very elegant. Well done!

We appreciate your feedback and compliment!

Page 4, column 2: “(first and fourth column)” “(third and fourth columns)”. At the beginning of the next page also change “(first column)” to “(third column)”.

Great that you spotted it, we modified the figure and adapted the caption but not all the text, which caused the problem. We corrected it, thank you.

Page 4, column 2: “... since a large portion of the observed reference import probability is zero due to a limited number of passengers.” Please expand this explanation.

We extended the explanation to “[...] since the estimated import probability is always nonzero, but a large proportion of the lowest reference import probabilities are zero due to the limited observation period and/or an insufficient number of departing passengers.”. We hope it is more clear now. In other words, in the year 2016 some countries did not exchange any passengers, which does not mean that they will never do so, but the reference import probability suggest exactly this. This mean that the reference import probability underestimates the lowest probability to 0.

Page 7, column 2: “showcasing its importance for epidemic related problem” “showcasing its importance for epidemic-related problems”.

Thank you, we corrected it.

Page 8, column 1: “can not” “cannot”.

Thank you, we corrected it in the whole document.

Page 8, column 1: “… or only these of the terminal nodes”. Do not understand what this sentence means.

Thanks for pointing out the unclear formulation. We changed it to: “[...] or only the exit probability of specific nodes (for details, see [...]”. Now it is clearer and instead of trying to explain which nodes are meant, we refer to the SI. For your interest, we mean the dead end nodes, i.e. all the nodes where the shortest path tree ends. “terminal nodes” was clearly too ambiguous.

Page 8, column 2: “increases” “increase”

Thank you, we corrected it.

Page 8, column 2: “However, we leave a possible extension” “However, we leave this as a possible extension”?

Thank you, we definitely missed some words.

Page S3: “less strong overestimated” “less strongly overestimated”

Thank you, we corrected it.

Page S3: “more likely return” “more likely to return”

Thank you, we corrected it.

Reviewer #2: In this study, the authors describe a model to compute import probabilities in the early stage of an epidemic based on the topological structure of the worldwide airport network.
The paper tackles an important research problem with significant implications for public health. It is clear that accurate estimates of import probabilities at the beginning of a pandemic represent an invaluable asset for policy makers.
Overall, the paper represents a relevant study whose results will be of interest to the readers of PLOS Computational Biology.

Before recommending the paper for publication, I highlight three main concerns with the hope that these will help improve the manuscript.

1-Background
I think the manuscript lacks some relevant context regarding the importance of estimating arrival times and importation probabilities in pandemic management.
First, I am a bit puzzled by the sentence in the abstract: “Accurate mechanistic models to estimate such risks are still lacking”. Here, it seems that we completely lack models to estimate importation risks in accurate ways, but I think this is not completely true.
For instance, the GLEAM model (Balcan, D., et al. 2010. Journal of Computational Science, 1, 132–145.) has been extensively used to compute importation risks for different outbreaks/pandemics. Just to give an example, please have a look at the platform https://epirisk.net.
Also, in the reference: Piontti, Ana Pastore Y., et al. "The infection tree of global epidemics." Network Science 2.1 (2014): 132-137, the authors provide a description of how to estimate the disease importation risk the worldwide air travel network.
Finally, importation probabilities and arrival times from Mexico have been used at the beginning of the 2009 H1H1 pandemic to estimate R0 (Tizzoni et al. BMC Medicine 2012) or to evaluate the effects of travel restrictions on the international spread of the 2014 WA Ebola epidemic (Poletto et al. Eurosurveillance 2014).
I understand the model presented in this paper extends and improves previous approaches, as it computes import probabilities considering all possible routes of travel, but still I think the Introduction could be rephrased to better contextualize the study.

We thank the referee for detailed comments and suggestions to the abstract and introduction. Especially for the abstract: being concise comes with a loss of some relevant information. As you highlighted, there are models as GLEAM that estimate the import probability. However, to our understanding, we would not

consider GLEAM as a “mechanistic model” because it is based on the gravity model, which is a fitted model that does not take the mechanisms of the passenger distribution into account. We understand that “mechanistic” is a very ambiguous, we changed in the abstract the following sentence
“As accurate mechanistic, parameter-free models are still lacking, we propose here the ``import risk'' model that defines an import probability by means of the effective-distance framework.”
→
“While there are data-fitted models available to estimate these risks, accurate mechanistic, parameter-free models are still lacking. Therefore, we introduce the 'import risk' model in this study, which defines import probabilities using the effective-distance framework.”

For the introduction, we restricted the literature background to the human mobility models, to which we count our model. From this point of view, we highlighted relevant basic models of human mobility and when referring to the disease dynamics on the human mobility networks, we restricted ourselves to reporting only those that motivate the use of the effective distance. Clearly we missed out some very relevant work to which you pointed us to, and that is mentioned in the new version of the manuscript. In the introduction we now refer to the multiscale mobility models (Balcan et al., PNAS 2009; Balkan et al., Journal of Computer Science 2010) and their application to disease outbreaks (H1N1: Tizzoni et al., BMC Medicine 2012; MERS: Poletto et al., Eurosurveillance 2014; Ebola: Poletto et al., Eurosurveillance 2014) in the 3rd paragraph of the introduction where we introduce the gravity models. To be explicit, we changed
“There exist mobility models that fit the OD matrix, i.e. a reference OD matrix is needed as for the gravity model \cite{Zipf1946, Cascetta1988, Lenormand2012, Abrahamsson1998, Barbosa2018}.”
→
“There are mobility models that fit the OD matrix, requiring a reference OD matrix as seen in the gravity model \cite{Zipf1946, Cascetta1988, Lenormand2012, Abrahamsson1998, Barbosa2018}. Additionally, some models integrate OD matrix-fitted models on a smaller scale with the OD matrix of the global air transportation network, creating a multiscale mobility network to represent all modes of transportation \cite{Balcan2009, Balcan2010}. Note that the multiscale mobility model has been successfully employed to analyze past pandemics \cite{Tizzoni2012, Poletto2014, Poletto2014a}.”

We did not include your proposed reference to Piontti. et al., Network Science (2014) in the introduction because it does not propose a way to compute the import probability but rather highlights the importance of the shortest path tree as infection tree in a pandemic. However, we find it is a fitting cite to highlight the information that is represented in WAN and used it in the section “Alternative models”.

2-The radiation model.
I see little justification to consider the radiation model as a good candidate model to represent worldwide movements at the intercontinental scale. The model was not originally developed to represent movements across countries, and especially when we consider countries in different continents. The theoretical framework of intervening opportunities is hard to apply to the case of the international air travel, given the presence of oceans, that are not populated. Its poor performance for this specific case does not surprise me.
In short: has ever the radiation model been used to model the WAN? If not, why using it here? What is the reason, besides its popularity? This choice would require more justification and results should be contextualized given the clear advantage of the gravity model.

We agree with the referee that the radiation model is not often applied on the WAN, but we believe mainly because the human mobility community focuses on commuter mobility and not as much on air passenger or global mobility. However, when mobility models are compared with each other the radiation model is often considered (e.g. Lenormand et al., Journal of Transport Geography 2016; Masucci et al., Phys. Rev. E 2013). In the air traffic community the gravity model is the standard, especially because its adaptability is highly valued and the model is not only fit to the population size but also to economic factors of the regions the airports are associated to, which makes the radiation model less useful to this community. Retrospectively, we agree with the referee that the poor performance is most likely due to the conceptualization of the radiation model based on opportunities ranked according to their distance which

is connected to a high loss in information (e.g. if an airport is on the other side of the ocean, it is just 1 rank away from the airport before the ocean starts). However, at least for us, it was not obvious since studies (Lenormand et al., Journal of Transport Geography 2016; Masucci et al., Phys. Rev. E 2013) suggested that the radiation model performs especially well at long ranges. Secondly, we did not use the classical radiation model but used it with a geodesic path and effective distance. For us, it seems also natural that the idea of job opportunities can be transferred to touristic opportunities. We made these points more clear in the “Alternative Models” section in the main text where we replaced

“It is derived from a mechanistic decision process and is in consequence parameter free, and therefore similar to our model.”
→
“Although past studies have found that the gravity model outperforms the radiation model at small scale \cite{Liang2013, Masucci2013, Lenormand2016}, especially the radiation model's good performance at the large scale \cite{Masucci2013, Lenormand2016} makes it an interesting model for mobility on the WAN. It was originally conceptualized for commuter flows \cite{Simini2012} where the surrounding populations serve as a proxy for possible job opportunities. By estimating an airport's population based on its outflow, we adjust the concept from job opportunities to tourism opportunities. Its derivation from a mechanistic decision process makes it parameter free, and therefore similar and a good comparison to our model.”

Additionally we added a paragraph in the discussion that highlights the likely reasons for the radiation models poor performance:

“The radiation model's poor performance can likely be attributed to its initial design, which focused on small-scale commuter flows driven by work opportunities \cite{Simini2012}, which shows that bottom-up approaches are often limited to their specific use case but can be adapted, such as the extended radiation model \cite{Yang2014}, which is no longer parameter-free and has similar performance to the gravity model \cite{Lenormand2016}. Interestingly, the radiation model is the only one that does not improve with inclusion of flight network information via the geodesic path or the effective distance (Fig. 4). The radiation model's insensitivity to network information can be attributed to the fact that it only extracts rank information from the distance data, resulting in a significant loss of information. The rank representation has the problem that airports that directly follow in their rank with respect to a source airport could be separated by a mountain range or ocean, i.e. the rank difference is minimal but the actual distance immense. This argument holds for any distance information.”

3- Real-world cases.
The theoretical analysis of the paper is well presented; however, I see one main limitation of the study in the fact that it does not consider any real-world epidemic to demonstrate the benefits/merits of the approach.

I think the paper would be significantly strengthened by an additional analysis considering a real epidemiological scenario, for instance the case of the COVID-19 pandemic.
What were the importation risks from Wuhan in December 2019? We know European countries were affected first, especially the case of Italy as one of the first countries to experience a major epidemic. Does the model capture the pattern of case importations we observed in the early stage of the pandemic? Does it provide insights that are new with respect to what could be done with simpler approaches?

We agree with the referee that the connection to real-world epidemics was in the initial manuscript too loose, because it was meant as a model description that should accompany another publication that used the import risk model in a world-wide epidemic model which estimates the pandemic potential of SARS-CoV-2 variants from noisy and limited data (Klamser and d’Andrea et al., Enhancing global preparedness during an ongoing pandemic from partial and noisy data, PNAS Nexus 2023). Thus, we intended to keep the results separated. However, we realized that in the light of model comparison an arrival time analysis of past pandemics would be no overlap at all, and thank the reviewer for making the point. We did not focus on the COVID-19 pandemic alone but wanted to include more outbreak scenarios,

because the arrival time is a noisy and probabilistic measure and our previous analysis use the import probabilities from all possible outbreak countries. Thus, we computed the correlation between the negative logarithm of the import probability estimates and the arrival times of the 2009 H1N1 outbreak, the 2019 COVID-19 outbreak and 18 of its variants (either variants of concern or variant of interest) that had enough data (spread to enough countries).
We added the section “Model comparison: Disease arrival times” with a new Figure (Fig. 6), a new paragraph in the discussion (5th paragraph), a part in the Methods, several figures and Tables in the SI and a part in the SI where we explore an alternative estimation of the arrival time via a log-cases fit (to reduce noise).
To answer your questions, we find that the models that use the effective distance perform extremely similar. That means, as long as the WAN information is considered, the course of a pandemic is represented realistically. We highlight in the discussion that the data is too sparse to recover the differences between import probability estimation models that we detected in comparison to the reference import probability data.

Additional to the comments of the reviewers, we corrected the parameters of the gravity model in use that are listed in the caption of Fig. S3 and corrected Fig. S3. The figure and the parameters referred to the best parameters of the non-symmetrized gravity models. However, in the main text, we refer to the symmetrized models.
