# Supplementary material for: Inferring country-specific import risk of diseases from the world air transportation network
Source: PLoS Comput Biol. 2024 Jan 24;20(1):e1011775. doi: 10.1371/journal.pcbi.1011775 (PMC10843136; doi:10.1371/journal.pcbi.1011775)
Supplement: Attachment — Submitted filename: PLOS COmputational Biology - Inferring country-specific import risk of diseases from the world air transportation network.docx [file pcbi.1011775.s002.docx]

**Inferring country-specific import risk of diseases from the world air transportation network**

The authors propose an “import risk” model, based on an import probability that is determined by the effective distance (according to which the higher the passenger flow in air transportation between two points, the closer the distance) and the (a) shortest possible path between two points. The length of the paths is in turn defined by means of a random walk on a tree. Theoretically, the model is very interesting, but its applicability is blurred because determining the effective distance in real life is extremely difficult due to the lack of a database that serves as a baseline.

I only have two major comments:

1. There is a problem with English grammar that makes the article difficult to read. I pointed to several instances in my minor comments but doubtlessly there are many more to be corrected. The article needs to go through a thorough grammar revision before acceptance.
2. The import risk model is very interesting mathematically. Nonetheless, the connection to import disease is loose. That is, despite suggesting the connection in the title and the abstract, the paper does little to justify this connection *and exploit it*. I think the paper is better fitted for PLOS One than for PLOS Computational Biology.

A more general comment: The symmetrization of the OD matrix is a very strong assumption that can lead to an overestimation of the import risk for countries with high levels of migration. For instance, it is not common for people from first-world countries to migrate to third-world countries, whereas the opposite is much more common, and this proportion does not seem negligible. Therefore, the import/export ratio is less than 1.

Minor comments:

Page 1, column 1: “as shown for the distribution of over 400 invasive species” 🡪 “as shown by the distribution of over 400 invasive species.”

Page 1, column 2: “Already the first plague pandemic that started 541 in the Nile” Is this B.C. or A.D.?

Page 1, column 2: “According to the effective distance, region B is closer to region A if the passenger flow from A to B is larger than to other destinations”. This clearly needs a third region C to make the comparison meaningful: B is closer to region A than to region C if the passenger flow from A to B is larger than that from B to C.

Page 1, column 2: “… from a specific source to target” 🡪 “from a specific source to a target”.

Page 2, column 1: “That means to exactly compute…” 🡪 “This means that to exactly compute…”

Page 2, column 1: “However, none of the above approaches use the effective distance with its validated link to disease propagation and none is based on a mechanistic distribution”. First, how is the link of effective distance to disease propagation “validated”? Because of the qualitative connection in the second paragraph? It sounds like this point needs at least some explanation and additional references. Second, what does “mechanistic distribution” mean? “Mechanistic” is again used in the next sentence, but its meaning is still unclear.

Page 2, column 1: “We find that the import risk model outperforms alternatives and only marginally improves if geodesic distance information is included”. Rewrite this sentence.

Page 2, column 2: Eq. (1) defines effective distance, but the definition of d_0 is extremely vague. From the previous explanation, it seems that it depends on i and j (the connecting flights between the two), so it is not constant. It is better to define it explicitly to avoid confusion.

Page 2, column 2: “(i) a random walk with exit probability”. Exit probability of/from/to…? Even though the concept is formally defined below, this heuristic must be clearer.

Page 2, column 2: “Applying a random walk is motivated by…” 🡪 “The use of a random walk is motivated by…”.

Page 3, column 1 (before eq(4)): “… all paths of length l to” 🡪 “all paths of length l as”.

Page 3, column 2: Eqs. (5) and (6), the core of the paper, are very elegant. Well done!

Page 4, column 2: “(first and fourth column)” 🡪 “(third and fourth columns)”. At the beginning of the next page also change “(first column)” to “(third column)”.

Page 4, column 2: “... since a large portion of the observed reference import probability is zero due to a limited number of passengers.” Please expand this explanation.

Page 7, column 2: “showcasing its importance for epidemic related problem” 🡪 “showcasing its importance for epidemic-related problems”.

Page 8, column 1: “can not” 🡪 “cannot”.

Page 8, column 1: “… or only these of the terminal nodes”. Do not understand what this sentence means.

Page 8, column 2: “increases” 🡪 “increase”

Page 8, column 2: “However, we leave a possible extension” 🡪 “However, we leave this as a possible extension”?

Page S3: “less strong overestimated” 🡪 “less strongly overestimated”

Page S3: “more likely return” 🡪 “more likely to return”
